# Effects of Empathic Paraphrasing – Extrinsic Emotion Regulation in Social Conflict

**DOI:** 10.3389/fpsyg.2012.00482

**Published:** 2012-11-12

**Authors:** Maria Seehausen, Philipp Kazzer, Malek Bajbouj, Kristin Prehn

**Affiliations:** ^1^Cluster of Excellence “Languages of Emotion,” Freie UniversitätBerlin, Germany; ^2^Dahlem Institute for Neuroimaging of Emotion, Freie UniversitätBerlin, Germany; ^3^Department of Psychiatry, Charité University Medicine Berlin, Campus Benjamin FranklinBerlin, Germany

**Keywords:** emotion regulation, empathy, social conflict resolution, paraphrasing, client-centered-therapy

## Abstract

In the present study, we investigated the effects of empathic paraphrasing as an extrinsic emotion regulation technique in social conflict. We hypothesized that negative emotions elicited by social conflict can be regulated extrinsically in a conversation by a listener following the narrator’s perspective and verbally expressing cognitive empathy. Twenty participants were interviewed on an ongoing or recently self-experienced social conflict. The interviewer utilized 10 standardized open questions inviting participants to describe their perception of the conflict. After each of the 10 descriptions, the interviewer responded by either paraphrasing or taking notes (control condition). Valence ratings pertaining to the current emotional state were assessed during the interview along with psychophysiological and voice recordings. Participants reported feeling less negative after hearing the interviewer paraphrase what they had said. In addition, we found a lower sound intensity of participants’ voices when answering to questions following a paraphrase. At the physiological level, skin conductance response, as well as heart rate, were higher during paraphrasing than during taking notes, while blood volume pulse amplitude was lower during paraphrasing, indicating higher autonomic arousal. The results show that demonstrating cognitive empathy through paraphrasing can extrinsically regulate negative emotion on a short-term basis. Paraphrasing led to enhanced autonomic activation in recipients, while at the same time influencing emotional valence in the direction of feeling better. A possible explanation for these results is that being treated in an empathic manner may stimulate a more intense emotion processing helping to transform and resolve the conflict.

## Introduction

Emotion regulation research to date has mainly focused on an individualistic point of view emphasizing control mechanisms in the individual, such as attention deployment, cognitive reappraisal, or the willful suppression of emotional expressions (Gross and Thompson, 2007; Butler and Gross, 2009; Rime, 2009). Compared to the abundance and sophistication of the research pertaining to classification schemes on such *intrinsic* regulation, systematic analysis of *extrinsic* emotion regulation and especially of controlled interpersonal affect regulation (i.e., the process of deliberately influencing the emotional state of another person, as opposed to non-conscious affect spreading) is still relatively sparse. Rime (2009), however, points out that an emotional experience is virtually indivisible of a social response, which in turn is bound to shape and modify the original emotion, so that emotion has to be regarded as a fundamentally interdependent process.

Niven et al. (2009) propose a classification system for controlled interpersonal affect regulation strategies, derived from Totterdell and Parkinson’s (1999) classification of strategies to deliberately improve one’s affect. Their final classification distinguishes between strategies used to improve versus strategies used to worsen others’ affect, and between strategies that engage the target in a situation or affective state versus relationship-oriented strategies. The technique of empathic paraphrasing, which is investigated in the present study, can be categorized as aiming at affect improvement and engagement within this classification framework. However, it also contains a relationship-oriented component, as empathic paraphrasing communicates interest and commitment in understanding the other’s perspective, thereby implying that their feelings are valid and worth listening to.

Empathy has been conceptualized in many different ways, usually involving a cognitive and an emotional component (Preston and de Waal, 2002; Lamm et al., 2007; Decety and Meyer, 2008). Cognitive empathy means the ability to take the perspective of another person and infer their mental state, while emotional empathy refers to the observer’s affective response to another person’s emotional state (Dziobek et al., 2008).

Paraphrasing or active listening (coined by Carl R. Rogers in Client-Centered-Therapy) is a form of responding empathically to the emotions of another person by repeating in other words what this person said while focusing on the essence of what they feel and what is important to them. In this way, the listener actively demonstrates that he or she can understand the speaker’s perspective (cognitive empathy). Rogers described empathy as the ability to sense the client’s private world as if it were one’s own, but without losing the “as if” quality (Rogers, 1951). Empathy is communicated through active listening, which in the Client-Centered approach aspires to evoke personal growth and transformation through providing a space of unconditional acceptance for the client. Rogers considered empathy, positive regard, and congruence both necessary and sufficient conditions for therapeutic change (Rogers, 1942, 1951).

This early notion on the importance of empathy for facilitating therapeutic change has gained ample empirical support over the last decades of research. How empathic a therapist is perceived to be has been identified as a critical factor for positive therapy outcome for both psychodynamically oriented and cognitive-behavioral psychotherapies (Bohart et al., 2002; Duan and Kivlighan, 2002; Orlinsky et al., 2004; Marci et al., 2007; Elliott et al., 2011; Norcross and Wampold, 2011). Based on a review of several studies Marci et al. (2007) describe a significant influence of perceived empathy on mood and general clinical improvement, even when controlling for other factors. Along this line, a meta-analysis conducted by Bohart et al. (2002) confirms a modest but consistent importance of empathy during psychotherapy. Zuroff et al. (2010) specifically examined the relationship between patient-reported measures of the three Rogerian conditions (positive regard, empathy, and genuineness) and therapeutic outcome, and found that patients whose therapists provided high average levels of the Rogerian conditions across all patients in their caseloads experienced more rapid reductions in both overall maladjustment and depressive vulnerability (self-critical perfectionism). Farber and Doolin (2011) conducted a meta-analysis on 18 studies also focusing on the effects of positive regard as defined by Rogers on treatment outcome, and found an aggregate effect size of 0.26, confirming a moderate influence of this factor.

The effectiveness of showing empathy on treatment success has also been assured within the field of medical care. Medical researchers have coined the term *clinical empathy*, which Mercer and Reynolds (2002) define as (1) understanding the patient’s situation, perspective and feelings (and their attached meanings), (2) communicating that understanding and checking its accuracy, and (3) acting on that understanding with the patient in a helpful (therapeutic) way. Hence, within the clinical setting empathy entails not only cognitive and affective components but also a behavioral component to communicate understanding to the patient, i.e., through active listening (Davis, 2009). Accordingly, the active demonstration of empathy has already been recognized as a crucial component of promoting cooperation in challenging situations within the field of clinical care. Halpern (2007) stresses that physicians who learn to empathize with patients during emotionally charged interactions can thereby increase their therapeutic impact. By the same token, a growing body of evidence demonstrates that empathic communication effectively helps patients through challenging and fearful situations, ranging from painful dental treatments over psychological problems to pandemic crisis (Cape, 2000; Reynolds and Quinn Crouse, 2008; Bernson et al., 2011). Neumann et al. (2009) reviewed prior empirical studies on clinical empathy and conclude that clinical empathy is a fundamental determinant of successful medical care, because “*it enables the clinician to fulfill key medical tasks more accurately, thereby achieving enhanced health outcomes*” (Neumann et al., 2009, p. 344).

In sum, the effectiveness of empathic communication as an extrinsic emotion regulation technique has already gained solid empirical support from psychotherapy and medical research. For the present study, social conflict was chosen as the context to examine the effects of empathic paraphrasing on emotion, for two reasons. Firstly, social conflict is often accompanied by intense emotions such as anger and hurt, and therefore lends itself easily to the investigation of extrinsic emotion regulation, without requiring artificial emotion induction in the laboratory. The setting of real-life social conflict renders it possible to work with “real” emotion, while at the same time concentrating on a non-clinical population. Secondly, empathic paraphrasing is used with vast prevalence within the field of conflict resolution. Paraphrasing is generally applied as one of the most important constitutional elements across all domains of conflict mediation (business mediation, family mediation, community mediation, victim-offender mediation, etc.). Hence, it seems expedient to take a closer look at the emotional effects of a technique so widely used within the context of its most common application.

Social psychology research offers evidence for a connection between dispositional affective empathy as well as dispositional perspective taking and adaptive social conflict behavior (Steins, 2000; Gehlbach, 2004; de Wied et al., 2007). However, there is hardly any research on the effects of *being treated* in an empathic manner (as opposed to feeling empathy oneself) on conflict behavior. Moran and Diamond (2008) report positive effects of therapist empathy on parent’s negative attitudes toward their depressed adolescent children. Being treated in an empathic way seems to help parents to also empathize with their children going through a rough time. This is an interesting finding, which contains parallels to social conflict situations and stimulates the question which emotional effects are triggered by being treated empathically, and how these emotional processes aid own empathic reactions toward others.

An interesting train of evidence regarding the socio-cognitive effects of being treated empathically is provided by research on interpersonal mimicry and language matching in social interaction. Numerous studies confirm that non-verbal interpersonal mimicry increases affiliation and positive social judgment as well as pro-social behavior not only toward the mimicker but also toward people not involved in the mimicry situation, indicating that being mimicked not only leads to an increased liking toward the interaction partner, but to an increased pro-social orientation in general (van Baaren et al., 2004; Ashton–James et al., 2007; Fischer-Lokou et al., 2011.; Guéguen et al., 2011; Stel and Harinck, 2011). This is true for the *mimickee* as well as the *mimicker* (Stel et al., 2008). Maddux et al. (2008) also report that strategic mimicry in negotiation abets more favorable negotiation outcomes, facilitating both individual and joint gains. This effect was mediated by higher levels of trust toward the mimicker. Ashton–James et al. (2007) tested several hypotheses on why mimicry promotes pro-social behavior and found that being mimicked during social interaction shifts self-construal toward becoming more interdependent and “other-oriented.” Additionally, mimicry strengthens one’s perception of interpersonal closeness with other people in general.

Correspondingly, language style matching, i.e., similarity in use of function words, has been found to predict relationship initiation and stability (Ireland et al., 2011). On a similar vein, according to the interactive-alignment account of dialog, the success of any given conversation depends on the extent of the conversation partners arriving at a common understanding of the relevant aspects of what they are talking about, i.e., a common situation model (Pickering and Garrod, 2004). Interlocutors tend to automatically align at different levels of linguistic representation, e.g., through repeating each other’s words and grammar (Garrod and Pickering, 2004). This alignment at low-level structure positively affects alignment of interlocutors’ situation models – the hallmark of successful communication – as people who describe a situation in the same way tend to think about it in the same way as well (Markman and Makin, 1998; Menenti et al., 2012). These findings strongly support the hypothesis that paraphrasing, which involves a certain degree of language matching and bears parallels to mimicry on a verbal level, administrates emotional and socio-cognitive effects on the person being paraphrased.

Regardless the impressive amount of research reviewed above, the specific dynamics of emotional response to empathic paraphrasing are yet largely unclear. Rime (2009) suggests that socio-affective responses such as comfort and empathy temporarily alleviate a narrator’s negative emotions and generate a deep feeling of relief. However, if no cognitive reframing and re-adjustment of goals, motives, models, and schemas occur, the alleviating effects of socio-affective responses can be expected to be only temporary, because the cognitive sources of the emotional unsettledness have not been transformed. Following this reasoning, the emotional effects of empathic paraphrasing should be expected to be short-lived. On the other hand, Rogers argued that receiving empathy and positive regard are necessary conditions for being able to revise overly rigid structures of the self and assimilate dissonant information and experiences (Rogers, 1942, 1951). Hence, empathic paraphrasing may initiate a cognitive-emotional process progressing in several stages, with emotional alleviation and an increased mental openness and disposition for cognitive restructuring possibly being the first one. In this respect, the present research makes a valuable contribution by moving beyond correlational designs to presenting the first experimental study assessing in detail the emotional effects of empathic paraphrasing in the context of social conflict, hopefully providing a useful basis for further analysis in future studies.

To investigate whether and how empathic paraphrasing in the context of a real-life social conflict extrinsically regulates emotion, we invited participants to an interview in which they were asked to talk about an ongoing or recently self-experienced social conflict with a partner, friend, roommate, neighbor, or family member. The interviewer responded to participants’ descriptions by either paraphrasing (experimental condition following half of the interview questions) or taking notes (control condition). We assessed valence ratings pertaining to participants’ current emotional state as well as skin conductance response (SCR), blood volume pulse (BVP), blood volume pulse amplitude (BVPamp), and heart rate (HR) as indicators of autonomous nervous system (ANS) activity during the interviews. We also recorded the interviews for documentation and analysis.

Psychophysiological and voice parameters have been proven to be reliable indicators for emotional responses (Scherer, 2003; Kushki et al., 2011). HR is regulated by sympathetic (increase) as well as parasympathetic (decrease) pathways of the ANS (Li and Chen, 2006; Kushki et al., 2011), and reflects autonomic arousal (Critchley, 2002) as well as emotional valence (Palomba et al., 1997). BVP is a measure of changes in the volume of blood in vessels and has been associated with affective and cognitive processing (Kushki et al., 2011). BVP amplitude has been found to be lower during episodes of increased sympathetic activity (Shelley, 2007) and has also been shown to decrease when feeling fear or sadness in several studies (Kreibig et al., 2007). SCR depicts changes in the skin’s ability to conduct electricity and is considered a sensitive psychophysiological index of changes in autonomic sympathetic arousal that are integrated with emotional and cognitive states. In addition, SCR reflects vicarious emotional responses to another’s affective state (pain), and is therefore also connected to empathy (Hein et al., 2011).

Based on the literature reviewed above, we hypothesized that empathic paraphrasing would lead to a reduction of negative emotion in the situation of talking about the conflict. Specifically, we expected valence ratings to be more positive after paraphrasing. Furthermore, we hypothesized that empathic paraphrasing would lead to lower autonomic arousal, reflected in psychophysiological measures and voice analysis.

## Materials and Methods

### Participants

Twenty healthy subjects [10 female; age: mean (M) = 27, standard deviation (SD) = 7.9] participated in this study. All participants were native German speakers, and had recently experienced a potentially ongoing social conflict with a partner, friend, roommate, neighbor, or family member. No conflicts involving physical or psychological violence were included in the study. Due to technical problems, SCR and voice data of four participants as well as BVP data of three participants were lost. Therefore, 20 participants entered the analysis of self-report data, 16 entered voice data analysis and analysis of SCR, and 17 entered analysis of HR and BVP.

The study was carried out in accordance with the Declaration of Helsinki and was approved by the ethical committee of the Charité University Medicine Berlin. All participants gave written informed consent prior to investigation and received payment for participation.

### Interview design and procedure

Participants were told that the study investigates emotion in social conflict, especially how emotions develop while speaking about a social conflict. The interviewer further informed participants that she would try to understand their perspective, and sometimes summarize what she understood so far, while at other times take notes to help her memorize certain things and have them present over the course of the interview.

Interviews consisted of 10 standardized open questions (e.g., “What exactly bothers you about the other person’s behavior?”). After the participant answered each question, the interviewer either paraphrased what had been said, or silently took notes (control condition). Following these paraphrasing interventions or control conditions, respectively, participants were asked to rate their current emotional state. In order to avoid confounding effects resulting from the content of the questions, as well as distortions due to emotional processing over the course of the interview, interventions, and control condition were given alternately during the interview. Half of all participants received an intervention (empathic paraphrasing) after the first question, a control intervention after the second question, and so forth; the other half received a control intervention first. All interviews were conducted by the same female interviewer, who had previously received 190 h of training in conflict resolution and has worked on cases in community mediation, business mediation, and family mediation over several years, applying empathic paraphrasing as one of the core techniques of conflict resolution.

Paraphrasing in the present study was implemented in such a way that after each narration the interviewer briefly summarized the facts of the narration and described her understanding of how the narrator felt, and why, and what she understood was important to the narrator regarding the situation described. To confirm the accuracy of her paraphrasing, the interviewer asked if her understanding was correct at the end of each paraphrase. An example of a paraphrase is given in the Appendix.

**Figure 1:**
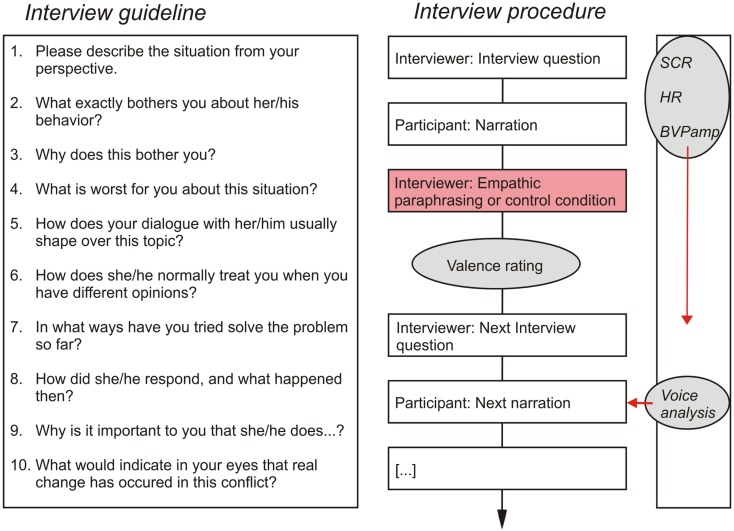
**Interview guideline and procedure**.

All interviews were audiotaped. Interview length was 30.16 min on average (SD = 11.03), depending on how extensively participants answered to the questions. Figure 1 depicts the interview questions as well as a schematic overview of the interview procedure and measurements.

### Data acquisition and analyses

Participants were asked to indicate their current emotional state (valence rating) on an eight-point Likert scale ranging from −4 to 4 (“How positive or negative do you feel right now?”) 10 times during the interview, following the interventions and control condition, respectively. Ratings were analyzed with two-tailed *t*-tests for repeated measures in IBM SPSS Statistics 20.

Skin conductance response and BVP were recorded continuously with a sampling frequency of 40 Hz using a commercial sampling device (*Biofeedback* 2000^X-pert^, Schuhfried GmbH, Austria) during the entire interview. Both interviewer’s and participant’s voices were recorded using Audacity 1.2.6 with a highly directional microphone (Shure, WH20 Dynamic Headset Microphone, IL, USA).

Skin conductance data was analyzed in LedaLab V3.3.1. Time frame of analysis was 25 s after the onset of the intervention or control condition. Within this interval, SCR was decomposed by continuous decomposition analysis (CDA; Benedek and Kaernbach, 2010). For each participant and interval, the maximum phasic activity was computed (with a minimum amplitude of 0.001 μS) and averaged for each participant across all intervals of both conditions).

Blood volume pulse and BVPamp were analyzed for intervals of 23 s after the onset of intervention or control condition using Matlab 7.1 (The Math-Works, Inc., MA, USA). Data were smoothed using a six point Gaussian filter. BVP was further used for extracting HR data through computing the inverse of the distance between successive peaks of the BVP signal in intervals larger than 0.4 s (Kushki et al., 2011). Mean SCR between both conditions (paraphrasing interventions and control conditions), BVP, BVPamp (in%), and HR (in beats per minute) were also analyzed with two-tailed *t*-tests for repeated measures in IBM SPSS Statistics 20. In addition, we compared BVP, BVPamp, and HR during the paraphrasing intervention and the interview question directly following the paraphrase, with a standard time frame of 4 s for the question phase.

Analysis of voice recordings was done with seewave in R statistics (Sueur et al., 2008). Using Audacity 1.2.6., intervals of speech for voice analysis were selected manually by listening to the recorded interviews and cutting out participants’ responses to each question – following an intervention or control intervention, respectively.

## Results

### Behavioral data

Valence ratings following paraphrasing revealed less negative feelings than ratings following the control condition [*t*(19) = 3.395, *p* = 0.003]. Effect size is *d* = 0.76 (Cohen’s *d* for repeated measures, calculated with pooled means and standard deviations).

**Figure 2:**
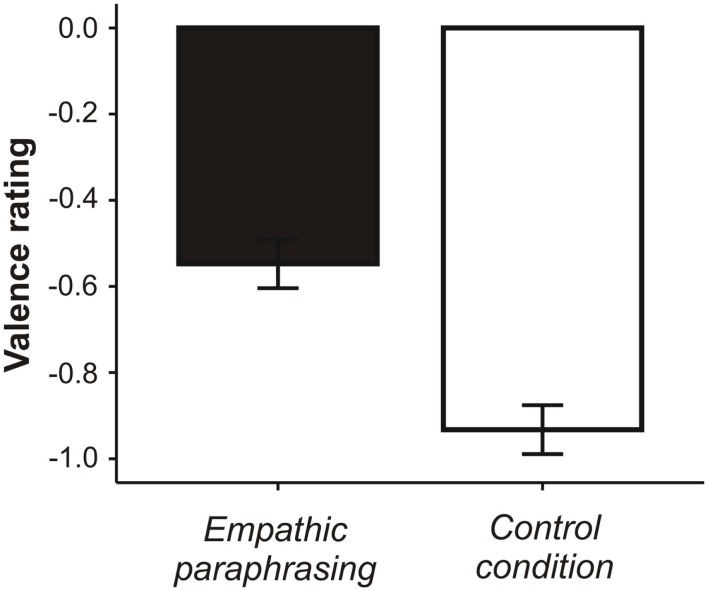
**Mean valence ratings (with standard error of the mean) after the empathic paraphrasing and control conditions**.

Differences in valence ratings over the conditions are shown in Figure 2.

**Figure 3:**
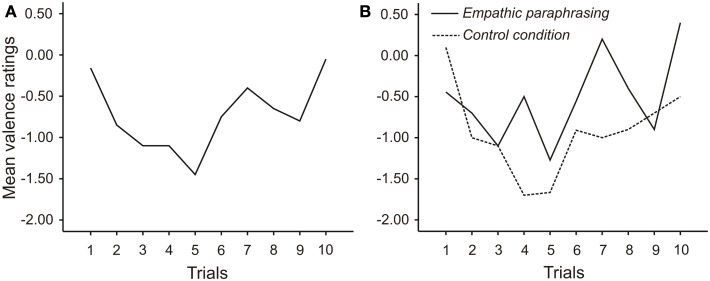
**Mean valence ratings over the course of the interview, averaged over both conditions (A) and split up into paraphrasing and control condition (B)**. At each of the 10 trials, 10 subjects received an intervention and 10 received a control intervention.

Time series plots over the entire course of the interview show a U-shaped trend in valence ratings over time, which is mainly due to ratings following the control condition (see Figure 3). However, a repeated measures ANOVA including sequence of intervention over time as an additional factor demonstrates that the effect of the intervention remains untouched by sequence [main effect of sequence *F*(4, 72) = 1.768; *p* = 0.145; main effect of intervention: *F*(1,18) = 11.400; *p* = 0.003 interaction intervention × sequence *F*(4, 72) = 1.489; *p* = 0.215].

### Psychophysiological data

**Figure 4:**
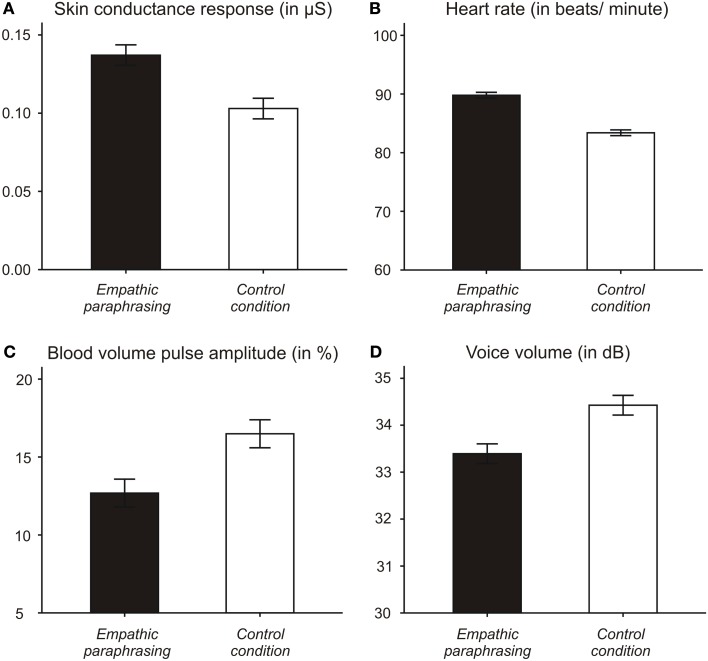
**Measures of sympathetic activation (mean values with standard error of the mean)**. **(A)** Skin conductance response (SCR; in μS), **(B)** Heart rate (in beats/minute), **(C)** Blood volume pulse amplitude (BVPamp in%), and **(D)** Voice volume (in dB) during empathic paraphrasing and control condition.

Two-tailed *t*-tests for repeated measures show that participants had a higher SCR during paraphrasing than during the control condition [*t*(15) = 2.589; *p* = 0.021]. Effect size is *d* = 0.65 (Cohen’s *d*). Complementary results were found in participants’ HR, which was also higher during paraphrasing than during the control condition [*t*(16) = 6.491; *p* = 0.000; effect size *d* = 1.57]. No significant differences between the conditions for BVP were found [*t*(16) = 0.22; *p* = 0.812]. However, there was a strong trend for mean BVPamp [*t*(16) = −2.119; *p* = 0.050; effect size *d* = 0.51], which was lower during paraphrasing than during taking notes. Comparing BVPamp during paraphrasing with the interview question directly following the paraphrase, we also found that BVPamp is lower during paraphrasing than during the following interview question [*t*(13) = 2.381; *p* = 0.033; effect size *d* = 0.64]. For HR and BVP, no such difference between paraphrase and subsequent interview question was found. Figure 4 illustrates differences in psychophysiological measures and voice intensity over the two conditions.

### Voice analysis data

Mean intensity/volume of participants’ voices was lower when they replied to an interview question following a paraphrase [*t*(15) = −2,466; *p* = 0.026; effect size *d* = 0.62]. There was no difference in mean fundamental voice frequency (F0) between the conditions [*t*(15) = 0.583; *p* = 0.568]. F0 range and F0 standard deviation did not differ between the conditions, either (see Table 1). However, speech rate and articulation rate showed trends for slower speech following paraphrasing [speech rate *t*(15) = −1.86; *p* = 0.082; articulation rate *t*(15) = −2.05; *p* = 0.059]. Cohen’s *d* yielded effect sizes of *d* = 0.47 for speech rate and *d* = 0.51 for articulation rate.

**Table 1:** **Means (M), standard deviations (SD), *t*-, *p*-, and *d*-values of all parameters in intervention and control condition**.

	Empathic paraphrasing		Control condition (taking notes)		*p*	*t*	Cohen’s *d*
	M	SD	M	SD			
Valence ratings (*N* = 20)	−0.55	1.10	−0.93	1.02	0.003**	3.40	0.76
**VOICE DATA (*N* = 16)**							
Volume (in dB)	33.40	3.57	34.43	2.83	0.026*	−2.47	0.62
Fundamental frequency (F0 in Hz)	249.09	8.26	249.33	8.41	0.568	−0.58	
Standard deviation F0	34.38	9.50	34.68	10.63	0.675	−0.43	
Range F0	315.98	30.24	312.75	47.56	0.745	0.33	
Speech rate	3.11	0.76	3.23	0.76	0.082	−1.86	0.47
Articulation rate	4.19	0.73	4.29	0.75	0.059	−2.05	0.51
**PSYCHOPHYSIOLOGICAL DATA (*N* = 17)**							
Skin conductance response (SCR in μS)	0.14	0.08	0.11	0.06	0.021*	2.59	0.65
Heart rate (HR in beats/minute)	89.79	8.94	83.39	10.89	0.000**	6.49	1.57
Blood volume pulse (BVP in%)	49.64	0.08	49.63	0.11	0.812	0.22	
Blood volume pulse amplitude (BVPamp in%)	12.68	6.93	16.49	12.65	0.050	−2.11	0.51

* and ** indicate significant findings.

Table 1 gives an overview of means and standard deviations of all psychophysiological, voice, and self-report parameters over the two conditions.

## Discussion

The aim of our study was to investigate the short-term emotional effects of empathic paraphrasing in social conflict. To achieve this, we conducted interviews on real-life social conflicts currently experienced by our participants. During the interview, paraphrasing was alternated with a control condition (taking notes). Emotional valence ratings were obtained after each intervention and control intervention and psychophysiological and voice recordings were executed continuously during the interviews. Our hypothesis was that paraphrasing would lead to more positive emotional valence and lower autonomic arousal. Viewing the results of our study as a whole suggests that empathic paraphrasing has a regulating effect on a narrator’s emotions, however, this effect seems to be more complex than originally expected. In sum, we found that participants felt better when the interviewer paraphrased their emotions and perceptions of the conflict. At the same time, and contrary to our expectations, SCR, HR, and BVP amplitude indicate higher autonomic activation during paraphrasing. Voice intensity as well as speech and articulation rate of participants on the other hand was lower when answering to a question following a paraphrase.

### Effects of paraphrasing on valence

The self-report ratings demonstrate that participants felt better after the interviewer had paraphrased what they had said. Also, the relatively high effect size suggests that this effect is strong and practically relevant. The interview itself also induced valence effects over time, insofar that participants experienced a decline in emotional valence in the middle of the interview, which recuperated toward the end of the interview. However, due to the alternation of intervention and control intervention, which was again alternated in sequence over participants, this trend does not affect the intervention effect.

This self-reported valence effect is consistent with participants’ lower voice intensity after paraphrasing compared to the control condition. Banse and Scherer (1996) have linked high voice intensity with negative affects or aggressive speaker attitudes, thereby suggesting a conjunction between high voice intensity and negative emotional valence. Conversely, speech and articulation rate are also slightly lower following an intervention, even though these effects are not statistically significant. Speech rate is defined as the number of spoken units (e.g., words/syllables) per unit of time (minute/second). It is calculated across continuous speech segments, which may include pauses, disruptions, or dysfluency. Articulation rate is an analogical measure based only on fluent utterances, excluding pauses, and dysfluency (Howell et al., 1999). Speech rate has been demonstrated to increase when experiencing anger or fear compared to neutral emotional states (Scherer, 1995; Rochman et al., 2008). Hence, the lower speech and articulation rates following paraphrasing also suggest that participants experienced less negative emotion after paraphrasing.

By the same token, HR was higher during paraphrasing than during the control condition, which according to Palomba et al. (1997) can also be interpreted as a valence effect. HR deceleration has been associated with negative emotional valence during presentation of unpleasant visual stimuli. In social tasks, HR acceleration has been measured in accordance with intensity of emotion, and to a lesser degree, with emotional valence (Palomba et al., 1997). Palomba et al. (1997) found significant differences in HR deceleration between positive, negative, and neutral visual stimuli, with positive stimuli producing the highest and negative stimuli the lowest HR. Hence, self-report data, voice data, and HR analysis all support the conclusion that emotional valence was positively influenced by offering cognitive empathy through paraphrasing. This effect of paraphrasing on valence bolsters Rime’s (2009) supposition that being treated empathically while socially sharing negative emotion produces a short-term alleviation of these negative emotions.

Interestingly, the positive impact of mimicry on social judgment mentioned in the introduction (i.e., promoting liking toward the mimicker) suggests the generation of positive emotion as a result of mimicry. This was not the case for paraphrasing in our study: valence ratings in the intervention condition center around the neutral. Nevertheless, it is still possible that paraphrasing led to an increased liking toward the interviewer, while overall affect was neutral. Social judgment was not assessed in the present study, hence, no direct comparison with mimicry is possible. However, it would be interesting to compare the effects of mimicry and paraphrasing on emotion in future studies, as well as to study verbal mimicry or matching more extensively in the context of distressing conversations such as social conflict discussions.

### Effects of paraphrasing on arousal

Skin conductance response, HR and BVP amplitude indicate a period of higher autonomic arousal while the interviewer paraphrased what participants had said, compared to taking notes on what they had said. Again, effects sizes of physiological measures suggest medium and in the case of HR, very strong, effects. This is surprising, as we presumed that the lower intensity of negative emotion induced by paraphrasing would be accompanied by lower arousal. Instead, paraphrasing apparently enhanced autonomic arousal. Quite conversely to psychophysiological data, the lower voice intensity following the intervention on the other hand suggests a calming effect of paraphrasing on autonomic arousal, as several studies on emotion and voice quality have associated high voice intensity with high sympathetic autonomic arousal emotions (Scherer, 2003). This apparent contradiction between voice data and psychophysiological data appears initially confusing, as vocal changes and changes in SCR both originate in mediated variation of HR, blood flow, and muscular tension caused by an arousing event (Duffy, 1932; Laver, 1968; Schirmer and Kotz, 2006).

However, this discrepancy can be explained by the fact that BVP and SCR were recorded *while* participants listened to the interviewer paraphrasing, whereas voice analysis was done on recordings of participants’ answers to the interviewer’s next question, *following* the paraphrase. Thus, the autonomic arousal induced by paraphrasing may already have subsided and passed into a calmer state at the time participants answered the next question. This possibility is difficult to double-check for SCR as this parameter is reactive to speech and will thus be higher while participants are talking, even though autonomic sympathetic arousal induced by the intervention might have diminished already. However, we reassessed this hypothesis using BVP, BVPamp, and HR data, comparing the paraphrasing phase with the subsequent question phase and found a confirming result for BVPamp, although not for the other two measures. Participant had a lower BVP amplitude while listening to the paraphrase compared to listening to the interview question asked in direct succession. This indicates a specific effect of paraphrasing on autonomic arousal, which is not induced by speech in general. It should also be noted that voice intensity following paraphrasing is significantly lower than voice intensity following the control condition. Hence, given the assumption made above is correct, participants’ autonomic arousal is first heightened by listening to the paraphrasing, and after a short period of time lowered to a level below the control state. This is a very interesting finding, for which two possible explanations should be considered.

Firstly, it is possible that empathic paraphrasing not only leads to a reduction of negative emotion in participants, but even induces positive emotions, such as happiness and relief about being listened to and validated. This would explain the initial higher autonomic arousal, which would in this case be due to a short-term experience of positive emotions, in accordance with Rime (2009) dissipating quickly. However, the behavioral data does not support this notion, as the valence ratings remain in the negative range of the scale even after paraphrasing, only approximating the neutral zero-point. Also, it should be noted that empathic paraphrasing is distinctly different from everyday forms of volunteering empathy or forms of social sharing of emotion as referred to by Rime. Paraphrasing does not offer sympathy or emotional empathy, but instead takes a purely cognitive road by demonstrating that the listener can understand the narrator’s perspective. It does not seem likely that this technique should have the same emotional effects as common social sharing responses such as offering sympathy.

Therefore, as an alternative explanation of our results, it is more conceivable that demonstrating cognitive empathy through paraphrasing temporarily leads to a heightened focus on and increased processing of negative emotion, which might eventually have a resolving effect on these emotions. This explanation seems probable considering the nature of paraphrasing, which entails repeating emotional narrations in a pointed way, thereby sharpening and clarifying the emotional experience. In a study on the relationship between therapist pre-session mood, therapist empathy, and session evaluation, Duan and Kivlighan (2002) found that intellectual empathy (demonstrating an understanding of the client’s perspective, i.e., empathic paraphrasing) was positively correlated with client-perceived session depth (power and value of the session), but not correlated with perceived session smoothness (comfort and pleasantness of the session). In a way, paraphrasing confronts people with what they are feeling, and thus can stimulate a deeper processing of negative emotion (depth), which temporarily involves higher autonomic arousal and may even be perceived as trying and hard work (smoothness), but eventually abets resolution of the emotional conflict. It however seems unlikely that this process advances automatically without fueling cognitive work such as reappraisal and re-adjustment of goals and schemas. Yet, the clarifying focus on one’s own emotion, accompanied by the non-judgmental stance of empathic paraphrasing might strongly push this process forward. This notion is in line with Rogers’ original claim to evoke personal growth and transformation in the client through empathic paraphrasing, thereby achieving therapeutic change (Rogers, 1942, 1951).

Also, considering the findings from mimicry and language matching research, which have demonstrated that being treated empathically on basal levels such as facial expression and language style promotes attitude and behavior change, it seems plausible that empathic paraphrasing may foster socio-cognitive processes in a similar direction. As paraphrasing contains a deliberate effort to verbally align with the narrator, it may generate a shared situation model and in this way promote successful communication. It would be interesting to consider if empathic paraphrasing, as it bears a certain resemblance to mimicry on a verbal level, can also stimulate pro-social behavior in the person being paraphrased; for instance a greater willingness to open up for the other party’s perspective on the conflict. This would strongly support the idea of paraphrasing stimulating a clearance of negative emotion.

There seems to be wide consensus between psychotherapists of different disciplines that psychotherapy benefits from an optimal level of arousal in the client, similar to the Yerkes–Dodson law, which posits an inverse U-shaped correlation between arousal and performance in complex tasks (Bridges, 2006). Markowitz and Milrod (2011) argue that emotional arousal is central for engaging the client in psychotherapy and making the therapeutic experience meaningful. They claim that the therapist’s ability to understand and respond empathically to negative emotional arousal should be considered the most important one of the common factors of psychotherapy. The therapist provides support and at the same time acts as a model, teaching the client to tolerate, verbalize, and integrate their feelings. Thus, negative feelings diminish and lose toxicity. In a similar vein, the traditional concept of the “corrective emotional experience” by Alexander and French (1946) describes the transformation of painful emotional conflicts as re-experiencing the old, unsettled conflict but with a new ending. This notion, which has gained ample empirical support, holds that processing emotional conflicts within a safe and empathic environment is necessary for therapeutic change (Bridges, 2006).

A resembling road is also pursued by acceptance and mindfulness-based interventions. Research on acceptance-based and mindfulness-based therapy has shown that accepting and mindfully observing negative emotions (instead of trying to suppress them) leads to the dissolution of these emotions (Eifert and Heffner, 2003; Arch and Craske, 2006; Hayes-Skelton et al., 2011). Czech et al. (2011) cite several experimental studies which have demonstrated that acceptance of negative emotion decreases distress and increases willingness to engage in challenging tasks. Empathic paraphrasing may have similar effects, as it essentially applies the principles of mindfulness and acceptance from the outside – through a listener who takes on an accepting role, thereby prompting the narrator in the same direction. Offering cognitive empathy through paraphrasing draws attention to emotions, non-judgmentally describes and accepts them, and is thus very similar to acceptance-based and mindfulness-based therapy. The central difference might be the locus of initiation of these processes, which in the case of empathic paraphrasing comes from somebody else. Comparing the effects of mindfulness and empathic paraphrasing and investigating the potential consequences of this difference on emotion processing and emotion regulation could be an interesting research focus for future studies.

### Limitations of the present study

A potential short-coming of the present study pertains to the nature of the control condition, which consisted of taking notes silently. It could be argued that, as only the experimental condition involved speech, the differences found might be due to a general effect of being spoken to, rather than to an isolated effect of empathic paraphrasing. However, it should be noted that within a social conflict situation, the content of a reply to emotional descriptions can never be perceived as completely neutral, and any control condition involving speech will induce emotional effects of its own, e.g., irritation or even anger caused by inapplicable verbal comments of the interviewer following participants’ emotional disclosure. The present control condition was deliberately chosen for providing a neutral baseline against which the effects of empathic paraphrasing can be tested before moving on to other modes of comparison.

An aligned point of concern might be that it cannot be ascertained how the control condition was perceived by participants. For instance, even though they were informed that the note-taking simply served the purpose of bolstering the interviewer’s memory during the conversation, some participants may still have worried about the notes containing subjective judgment. This would most likely induce stress and add an emotional bias to the control condition. In this case, however, one would expect an increase in autonomic responses during the control condition, which did not occur. Still, considering these shortcomings of the control condition, the results need to be reproduced with varying kinds of control conditions involving speech before they can be viewed as definite.

It should also be mentioned that this study focused exclusively on short-term emotional reactions to paraphrasing, in order to obtain a constitutional data base illustrating the regulatory effect of this communicational technique. Our results suggest that in addition to influencing immediate emotional valence, paraphrasing sets in motion an initially arousing process of coping with negative emotions associated with the social conflict, which eventually may lead to resolving these emotions. However, as we did not assess longitudinal measures pertaining to the emotions associated with the social conflicts in question, this conclusion has to remain speculative until backed up by further research.

Finally, the relatively small sample size of the study makes it prone to distortions from individual variations and gender differences, e.g., in emotion expression. Again, replication of the results based on larger groups of study participants is called for.

### Conclusion and directions for future research

The present study provides first experimental evidence that offering cognitive empathy through paraphrasing extrinsically regulates emotion in social conflict. Paraphrasing led to less negative feelings in study participants, while at the same time inducing higher autonomic arousal, which subsided after a short period of time. A possible explanation for these findings is that empathic paraphrasing stimulates an increased and focused processing of negative emotion in social conflict, and thus may contribute to resolving these emotions.

Future studies investigating the emotional effects of demonstrating cognitive empathy may further scrutinize the short- and long-term effects empathic paraphrasing has on arousal, and test the hypothesis that paraphrasing induces a cognitive-emotional process which facilitates the resolution of negative emotion in social conflict. Also, it would be interesting to investigate the dynamics of this process more closely and identify factors necessary for its successful development. Presently, we are working on a neuroimaging paradigm designed to overcome some of the above mentioned shortcomings and further explore the effects of empathic paraphrasing on the disposition to consider other people’s perspective in social conflict.

## Conflict of Interest Statement

The authors declare that the research was conducted in the absence of any commercial or financial relationships that could be construed as a potential conflict of interest.

## Appendix

### Example of a paraphrasing sequence

Interviewer: “What is worst for you about this situation?”

Narrator: “The worst thing is not knowing what happens now, well, this uncertainty. I mean, there is a problem, I have to make sure the rent is being paid, because in the end I am responsible, because I am in the rental agreement…and then – not being able to deal with that situation, not being able to act, because I just don’t know what is going to happen. The worst…now I am not so sure anymore, what was worst about it – well, also interpersonally it was very disappointing, because after all I took care of everything, voluntarily, and…I mean, when she is acting this way now, that is also a lack of recognition for what I do, what I accomplish. For my whole courtesy. What aggravates things is that is was clear from the beginning that she does not do so well financially, but urgently needed an apartment, and I let her move in with me to help her. And that is something that is…not being trampled under her feet…but you notice that there is a lack of recognition. Well, I think this second issue is worse than the first one.”

Interviewer: “So it is a combination, is it? For one, this thing, that in some way your existence is on stake here, that you are saying, this uncertainty is hard to bear – that you do not know how the rent is going to come around in the future. And then also the interpersonal issue, that you are saying you are disappointed of her, because you helped her, and in return you get this now, right? Especially the lack of recognition, the interpersonal treatment is what is worst – did I understand that correctly?”

Narrator: “Yes.”
